# Recurrent Seizure-Triggered Takotsubo Syndrome With Phenotypic Switching and Rapid Functional Recovery: A Case Report

**DOI:** 10.7759/cureus.105990

**Published:** 2026-03-27

**Authors:** Jonathan Moyambi, Alfakihi Ahmed Said IsmaIl, Fervent Ndukute, Adolphe M Kasongo

**Affiliations:** 1 Cardiology, Ibn Rochd University Hospital, Faculty of Medicine and Pharmacy, Hassan II University, Casablanca, MAR; 2 Cardiology, Sud Francilien Hospital, Corbeil-Essonnes, FRA

**Keywords:** apical ballooning, cardiac magnetic resonance, epilepsy, phenotypic switching, recurrent takotsubo syndrome, seizure-triggered cardiomyopathy, takotsubo syndrome

## Abstract

Takotsubo syndrome (TTS) is an acute cardiomyopathy characterized by transient left ventricular systolic dysfunction, often mimicking acute coronary syndrome. Current pathophysiological concepts emphasize sympathetic overactivity and catecholamine excess as major determinants of myocardial stunning, and experimental models further suggest that high epinephrine concentrations may induce a beta-2 adrenoceptor Gs-to-Gi signaling switch, contributing to severe but reversible myocardial dysfunction. Neurological triggers, particularly epileptic seizures, are increasingly recognized and may be associated with distinctive clinical profiles, arrhythmic complications, recurrence, and variable ventricular morphology.

We report the case of a 57-year-old woman with epilepsy and chronic obstructive pulmonary disease who presented with acute chest pain immediately after a generalized tonic-clonic seizure. She had experienced a previous TTS episode 11 months earlier, documented as a mid-ventricular variant with a left ventricular ejection fraction (LVEF) of 50%. During the recurrent episode, electrocardiography showed sinus rhythm without ST-segment elevation, with known anteroseptal T-wave inversion. Serial laboratory testing demonstrated a relatively modest peak high-sensitivity troponin T level of 140 pg/mL despite severe left ventricular dysfunction, together with markedly elevated N-terminal pro-B-type natriuretic peptide. Transthoracic echocardiography showed an LVEF of 20%-25%, with apical hypokinesia extending to the adjacent mid-ventricular segments and relative basal hypercontractility. Coronary angiography showed no acute culprit lesion and was unchanged from the prior examination. Left ventriculography confirmed apical ballooning with mid-ventricular extension. By day 3, LVEF had improved to 52%, while cardiac magnetic resonance imaging showed normalized wall motion with mild residual myocardial edema. This case highlights recurrent seizure-triggered TTS with documented phenotypic switching and rapid functional recovery, emphasizing the value of multimodality imaging and coordinated neuro-cardiac management.

## Introduction

Takotsubo syndrome (TTS) is an acute cardiomyopathy characterized by transient left ventricular systolic dysfunction and a clinical presentation that often mimics acute coronary syndrome, with chest pain, electrocardiographic abnormalities, and troponin elevation despite the absence of an acute culprit coronary lesion [1]. Consensus documents have further clarified its diagnostic criteria, the spectrum of ventricular phenotypes, and the central role of multimodality imaging in confirming the diagnosis and excluding important differential diagnoses, particularly myocarditis [2]. Current pathophysiological concepts emphasize sympathetic overactivity and catecholamine excess as major determinants of myocardial stunning and clinical variability [3]. Experimental models further suggest that high epinephrine concentrations may induce a β2-adrenergic signaling switch from Gs to Gi coupling, resulting in a transient negative inotropic effect that may help explain the combination of severe but reversible left ventricular dysfunction observed in TTS [2,3].

Neurological triggers occupy a distinctive place within the TTS spectrum and may differ mechanistically from classical emotionally triggered forms. In seizure-related TTS, the epileptic event may induce a postictal autonomic storm characterized by abrupt sympathetic discharge, transient hypoxemia, and marked neurohumoral activation [3-5]. Dysfunction of the central autonomic network, including the insular cortex, amygdala, hypothalamus, and brainstem, may contribute to excessive cardiac sympathetic output, arrhythmic vulnerability, and regionally heterogeneous ventricular dysfunction [4,5]. These mechanisms may account for the distinctive clinical profiles reported in neurologically triggered TTS [4]. Recurrence is uncommon but well documented and may occur with morphological variation between episodes, supporting the concept of phenotypic switching and persistent susceptibility of the neuro-cardiac axis [6-8].

We report the case of a woman with epilepsy who developed recurrent seizure-triggered TTS associated with phenotypic switching between a first mid-ventricular episode and a second apical episode extending to the mid-ventricular segments, together with very rapid functional recovery.

## Case presentation

A 57-year-old woman with a history of epilepsy diagnosed in 2019 and chronic obstructive pulmonary disease, receiving fluticasone furoate/vilanterol 92/22 µg (Relvar Ellipta), lamotrigine 150 mg/day, aspirin, and statin therapy for previously documented non-obstructive coronary artery disease in 2024. She was admitted for acute chest pain occurring immediately after an epileptic seizure. Her neurological history was notable for a previous episode of status epilepticus, initially treated with levetiracetam and subsequently switched to lamotrigine 150 mg. A prior brain magnetic resonance imaging study had shown an interhemispheric cerebellar cystic lesion.

From a cardiovascular standpoint, the patient had experienced a first episode of TTS in November 2024. Ventriculography at that time demonstrated a mid-ventricular variant, with dyskinesia of the mid-ventricular segments and relative hyperkinesia of the basal and apical segments. Left ventricular ejection fraction (LVEF) was estimated at 50% (normal range in women: 54%-74%). Coronary angiography performed during that first episode showed an intermediate single-vessel lesion of the left anterior descending artery that was not functionally significant, with a fractional flow reserve of 0.86 (functional significance threshold: ≤0.80), as well as a long non-significant stenosis of the right coronary artery.

**Figure 1:**
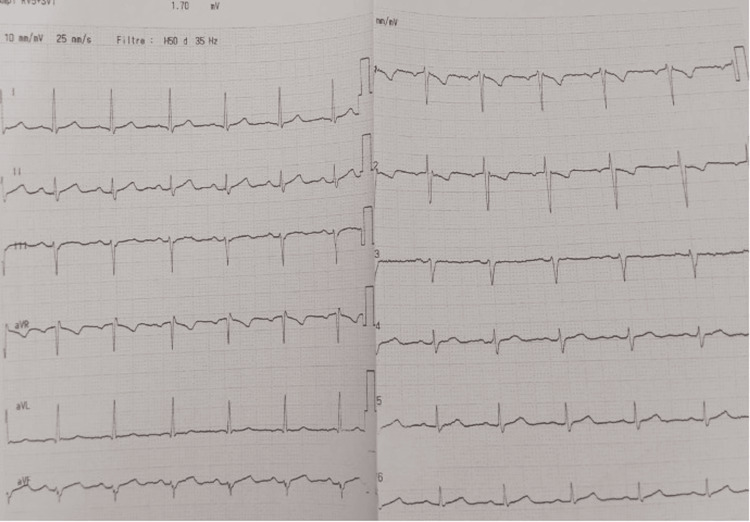
A 12-lead electrocardiogram showed sinus rhythm without ST-segment elevation, with known anteroseptal T-wave inversion and no QT prolongation.

Eleven months later, in October 2025, the patient presented with a new episode beginning during the night with a generalized tonic-clonic seizure lasting approximately two minutes, according to her husband, and associated with urinary incontinence, followed by retrosternal chest pain radiating to the neck. On admission, she was hemodynamically stable, with a heart rate of 92 beats/min, blood pressure of 110/81 mmHg, and oxygen saturation of 94% on room air. A 12-lead electrocardiogram showed sinus rhythm without ST-segment elevation, with known anteroseptal T-wave inversion and no QT prolongation (Figure 1).

**Figure 2:**
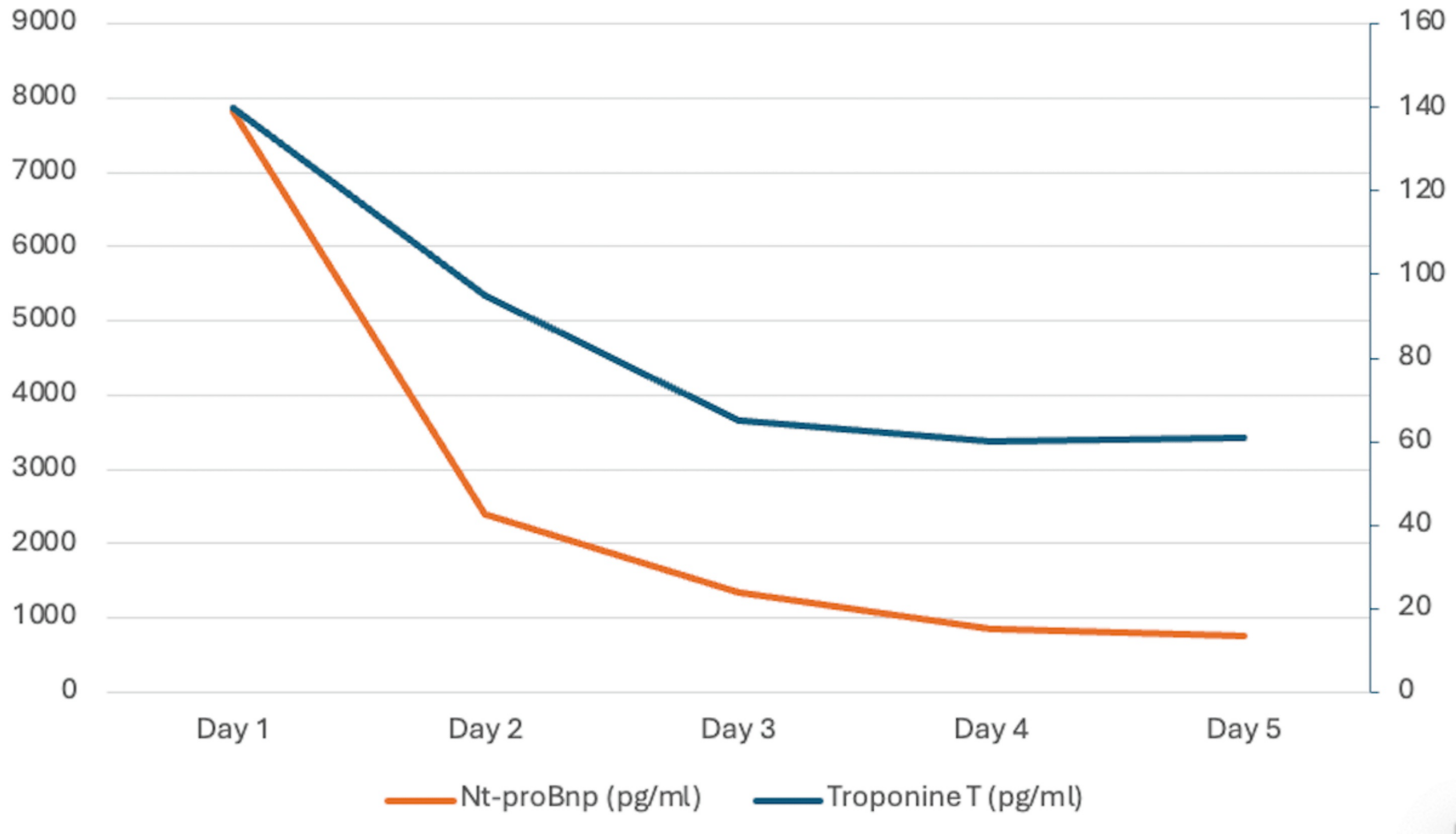
Time course of high-sensitivity troponin T and NT-proBNP levels during hospitalization. Serial measurements of cardiac biomarkers during hospitalization showing a progressive decline in high-sensitivity troponin T and N-terminal pro-B-type natriuretic peptide (NT-proBNP) levels from day 1 to day 5. High-sensitivity troponin T decreased from 140 pg/mL on day 1 to 61 pg/mL on day 5 (upper reference limit: 14 pg/mL), while NT-proBNP declined from 7,818 pg/mL to 754 pg/mL (rule-out threshold for acute heart failure: <300 pg/mL). This dissociation between modest troponin elevation and markedly elevated NT-proBNP is consistent with acute myocardial stunning in Takotsubo syndrome. Image was created using Microsoft Word (Microsoft® Corp., Redmond, WA).

Serial laboratory testing demonstrated elevated cardiac biomarkers with a downward trend: high-sensitivity troponin T was 140 pg/mL on day 1, 95 pg/mL on day 2, 65 pg/mL on day 3, 60 pg/mL on day 4, and 61 pg/mL on day 5 (upper reference limit: 14 pg/mL). N-terminal pro-B-type natriuretic peptide was 7,818 pg/mL on day 1, decreasing to 2,400 pg/mL on day 2, 1,347 pg/mL on day 3, 856 pg/mL on day 4, and 754 pg/mL on day 5 (rule-out threshold for acute heart failure: <300 pg/mL) (Figure 2).

**Figure 3:**
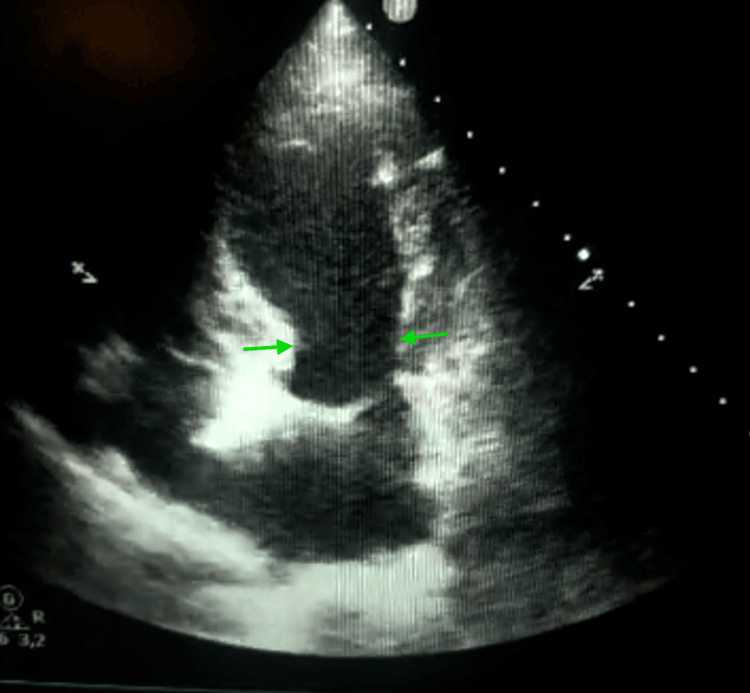
Apical four-chamber transthoracic echocardiographic view obtained on admission. Apical four-chamber transthoracic echocardiographic view obtained at admission, demonstrating severe left ventricular systolic dysfunction with apical akinesia extending to the adjacent mid-ventricular segments, relative basal hypercontractility, and a visually estimated left ventricular ejection fraction of 20%-25%. The arrows indicate the hypercontractile basal segments.

Transthoracic echocardiography performed on day 0 showed severe left ventricular systolic dysfunction, with a visually estimated LVEF of 20%-25% (normal range in women: 54%-74%) on emergency assessment, showing akinesia of the apex with extension to the adjacent mid-ventricular segments and relative basal hypercontractility, consistent with an apical Takotsubo phenotype with mid-ventricular extension (Figure 3).

**Figure 4:**
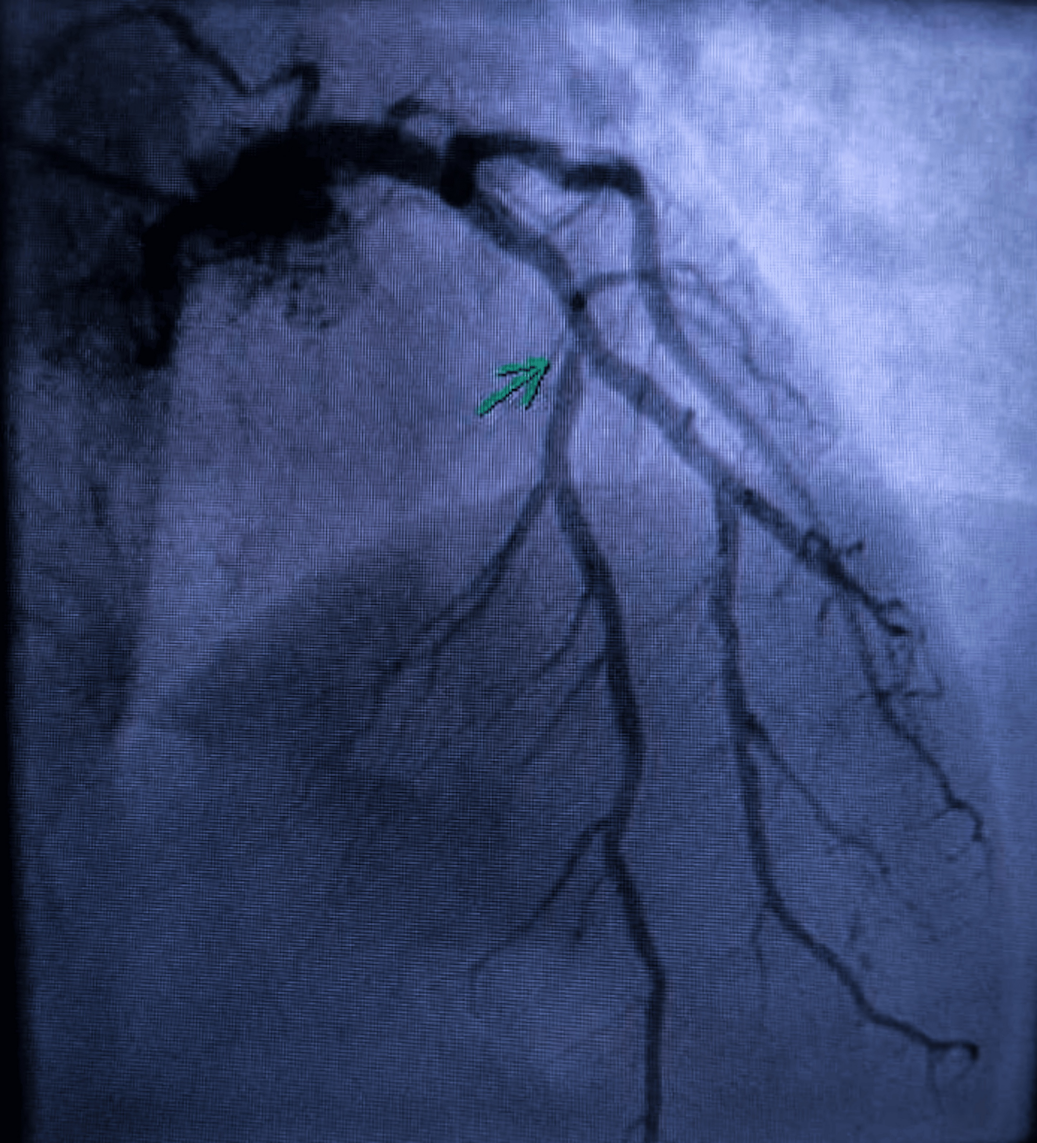
Coronary angiography of the left anterior descending artery in the right anterior oblique (RAO) 10°/40° cranial view. The arrow indicates an intermediate lesion.

**Figure 5:**
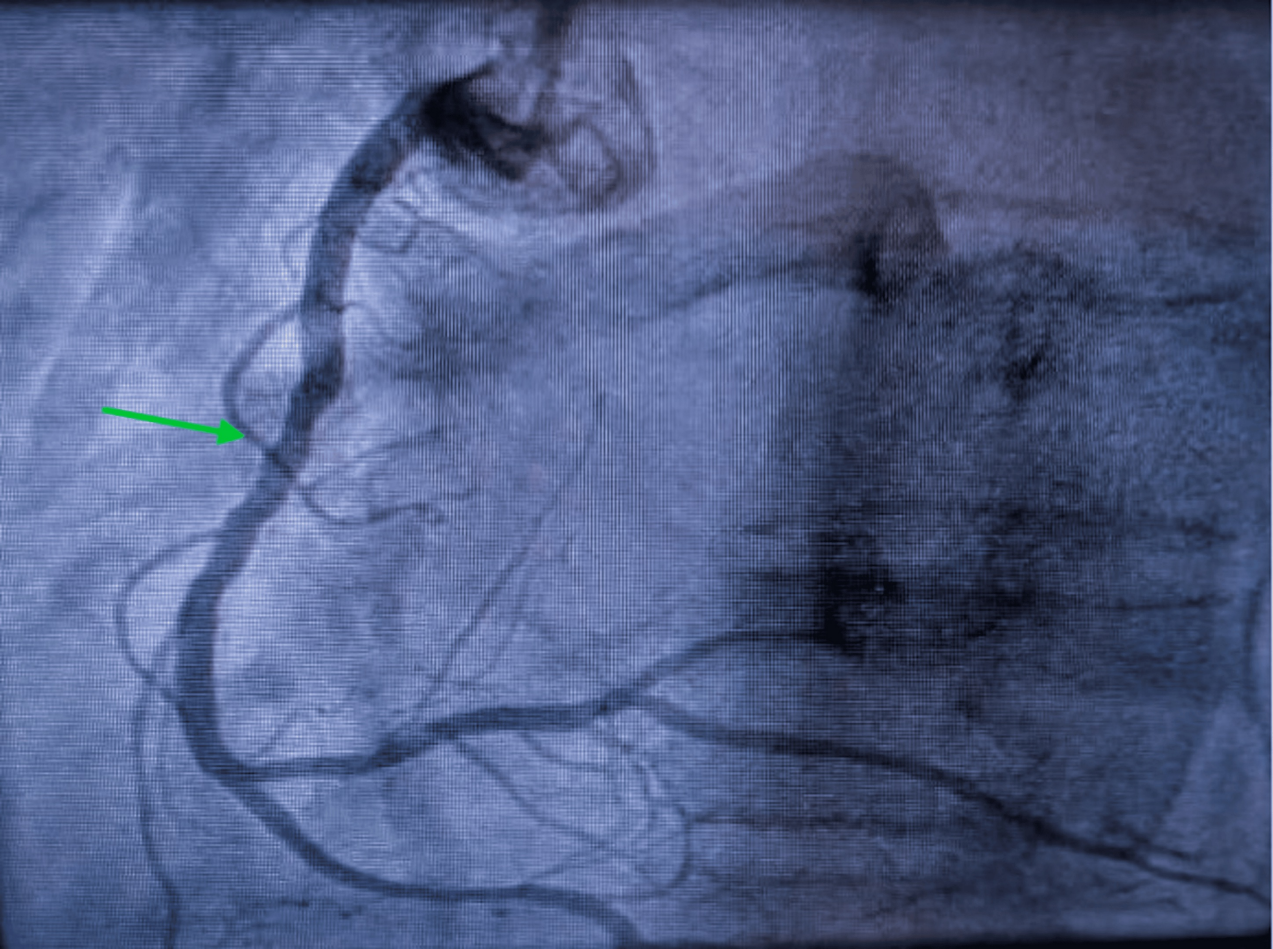
Coronary angiography of the right coronary artery in the left anterior oblique (LAO) 30° view. The arrow indicates a long, non-significant stenosis involving segment 2.

Given the pseudo-acute coronary syndrome presentation, coronary angiography was performed on day 1. It showed no acute culprit coronary lesion and appeared unchanged compared with the previous examination (Figures 4-5).

**Figure 6:**
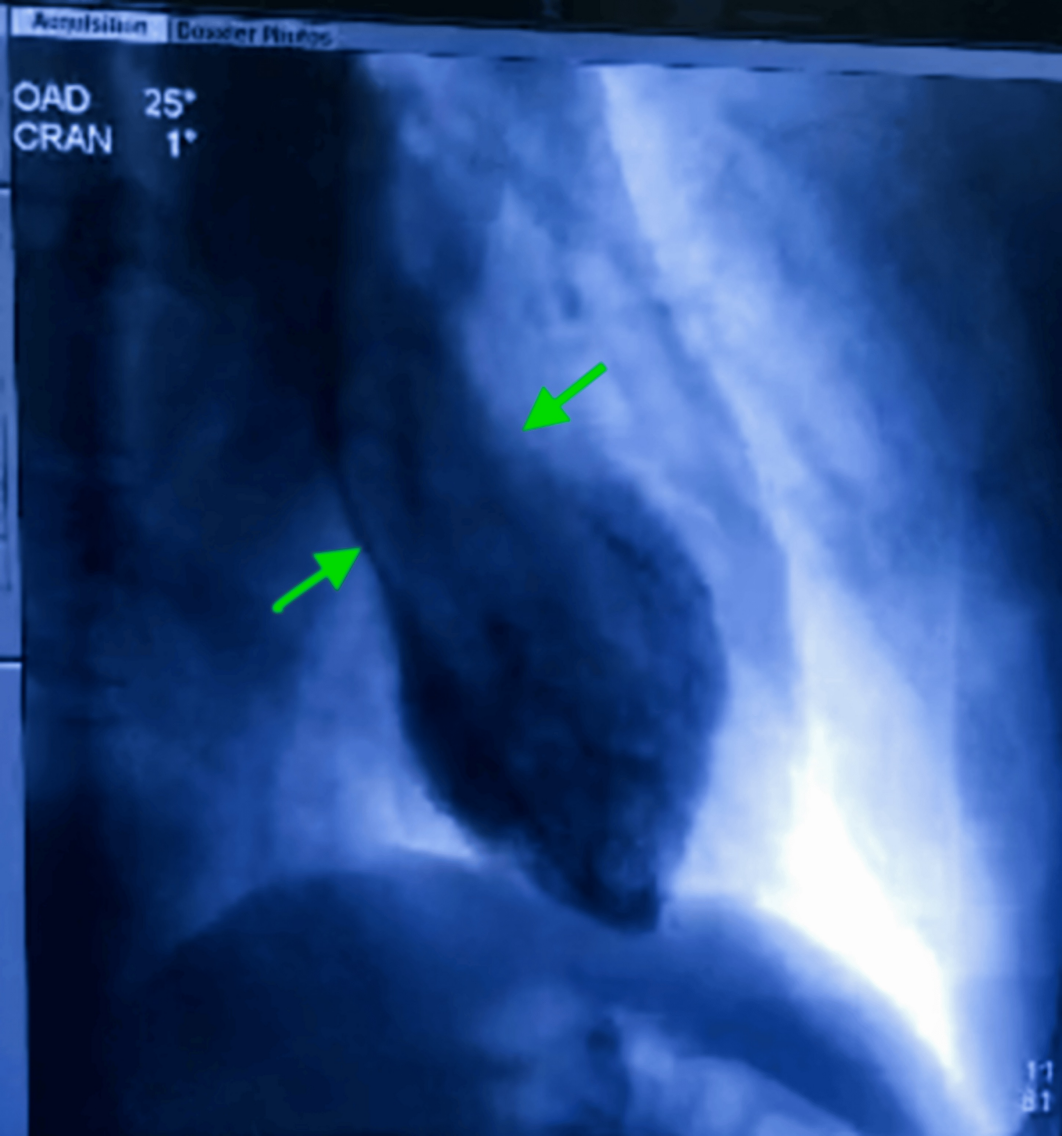
Left ventriculography in the 25° right anterior oblique (RAO) projection demonstrating the classic Takotsubo pattern, characterized by apical ballooning, also known as the “octopus trap” configuration, as indicated by the arrow.

Left ventriculography in the 25° right anterior oblique projection demonstrated the classic Takotsubo pattern, characterized by apical ballooning extending to the mid-ventricular segments, with relative basal hypercontractility (Figure 6).

**Figure 7:**
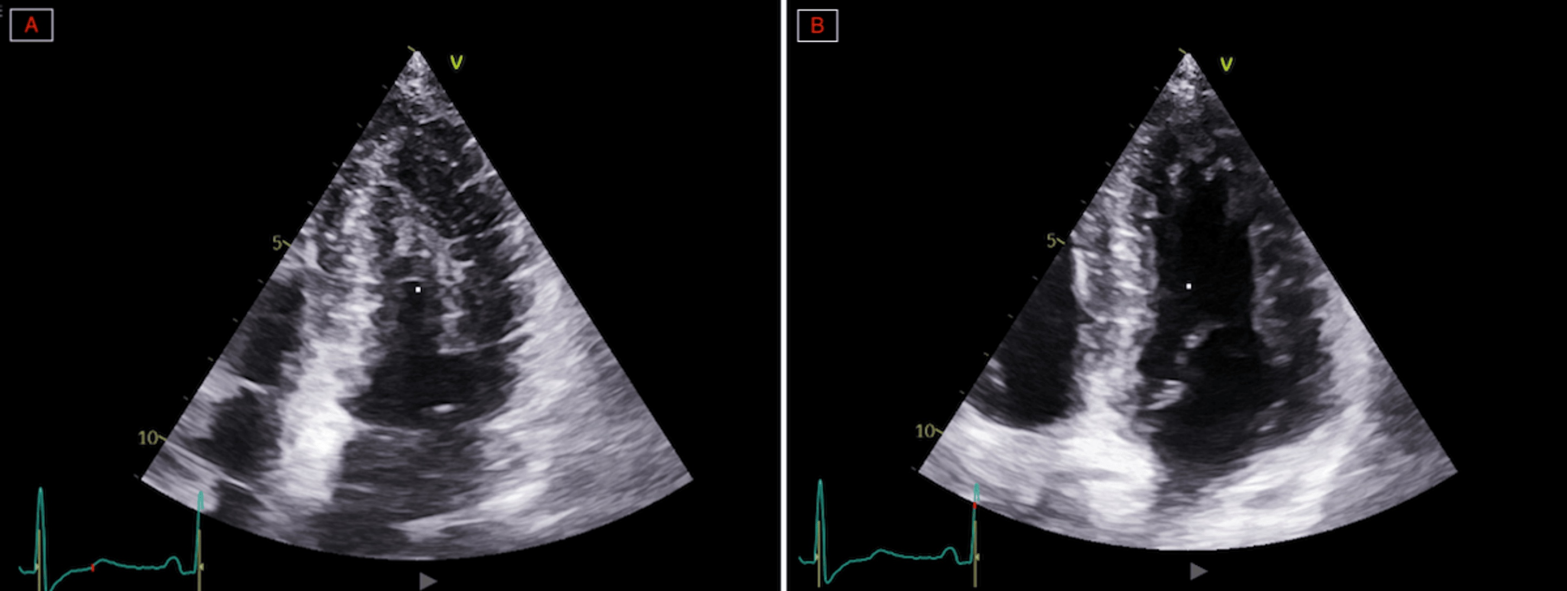
Apical four-chamber transthoracic echocardiographic views focused on the left ventricle on day 3. (A) End-diastolic frame. (B) End-systolic frame. These images demonstrate rapid recovery of left ventricular systolic function, with improvement of left ventricular ejection fraction to 52% and normalization of segmental wall motion.

Hemodynamic evolution was favorable. On day 3, follow-up transthoracic echocardiography showed rapid recovery of left ventricular function, with an LVEF of 52% (normal range in women: 54%-74%) (Figures 7A-7B).

**Figure 8:**
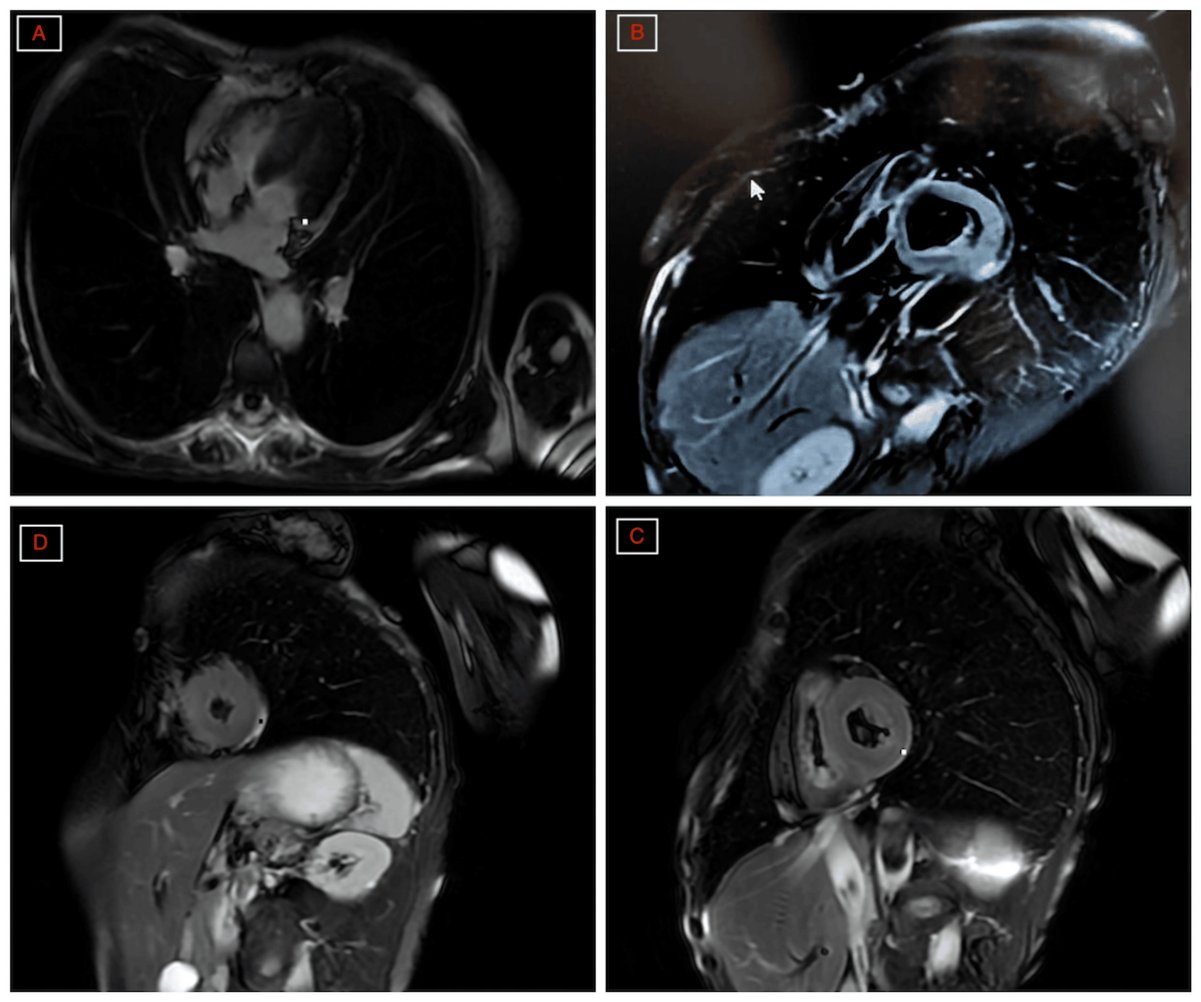
Cardiac magnetic resonance imaging findings on day 3. Cardiac magnetic resonance imaging findings on day 3. (A) Four-chamber late gadolinium enhancement (LGE) view showing no myocardial LGE. (B) T2-weighted black-blood fat-saturated short-axis image at the basal level, without significant myocardial edema. (C) T2-weighted black-blood fat-saturated short-axis image at the mid-ventricular level, showing mild myocardial edema. (D) T2-weighted black-blood fat-saturated short-axis image at the apical level, also showing mild myocardial edema.

Cardiac magnetic resonance (CMR) imaging performed at the same time showed normalized segmental wall motion with persistent mild residual myocardial edema, consistent with TTS in the subacute recovery phase; LVEF was 62% (normal range in women: 54%-74%) on CMR imaging (Figures 8A-8D).

Telemetry revealed runs of non-sustained ventricular tachycardia, leading to the initiation of bisoprolol 2.5 mg/day. In consultation with the neurology team, lamotrigine was increased from 150 mg/day to 200 mg/day to optimize seizure control. The subsequent course was favorable, without recurrent chest pain or hemodynamic instability. The patient was discharged on day 5 with continuation of antiepileptic therapy and planned cardiology and neurology follow-up.

The clinical timeline was as follows: epilepsy was diagnosed in 2019, and her history later became notable for status epilepticus. In November 2024, she experienced her first episode of TTS with a mid-ventricular phenotype and an LVEF of 50%, while coronary angiography showed non-obstructive coronary lesions. In October 2025, 11 months later, she developed a nocturnal epileptic seizure followed by chest pain. On day 1, high-sensitivity troponin T was 140 ng/L, N-terminal pro-B-type natriuretic peptide was 7,818 pg/mL, and transthoracic echocardiography showed an LVEF of 20%-25% with apical and mid-ventricular akinesia and basal hypercontractility. On day 1, coronary angiography showed no acute culprit lesion. On day 3, transthoracic echocardiography showed recovery of LVEF to 52%, while CMR imaging showed normalized wall motion with mild residual myocardial edema. During hospitalization, the patient developed non-sustained ventricular tachycardia, and bisoprolol 2.5 mg/day was initiated. Given the marked cardiac consequences of the seizure, the antiepileptic regimen was reassessed with the neurology team. As no recent drug interaction or nonadherence was identified, lamotrigine was increased from 150 mg to 200 mg/day to optimize seizure control. The patient was discharged on day 5 with planned cardiology and neurology follow-up.

## Discussion

The initial presentation combined chest pain, electrocardiographic abnormalities, and troponin elevation, warranting a structured diagnostic approach aimed at ruling out acute coronary syndrome in accordance with contemporary guidelines [9]. This presentation is typical of TTS, whose initial phase is often indistinguishable from acute coronary syndrome [1,2]. In our case, transthoracic echocardiography performed on day 0 played a central role in the diagnostic workup by immediately demonstrating severe left ventricular systolic dysfunction, with a visually estimated LVEF of 20%-25%, and a regional wall motion abnormality characterized in apical four-chamber and two-chamber views by apical akinesia extending into the adjacent mid-ventricular segments, with relative basal hypercontractility. This pattern was further confirmed by left ventriculography performed during the 2025 episode, which demonstrated an apical Takotsubo phenotype with ballooning of the apex extending into the adjacent mid-ventricular segments, while the basal segments remained relatively preserved and hypercontractile. Importantly, this contrasted with the ventriculographic findings from the 2024 episode, which had shown a mid-ventricular phenotype characterized by dyskinesia of the mid-ventricular segments with relative hyperkinesia of both the apical and basal segments. Taken together, these findings support true phenotypic switching between two distinct Takotsubo morphologies in the same patient.

The close temporal relationship between the epileptic seizure and the onset of cardiac symptoms is consistent with the mechanisms described in the heart-brain interaction in epilepsy, including peri-ictal dysautonomia, catecholamine overload, transient hypoxia, and reversible myocardial stress [5]. In our patient, the apical ballooning pattern with extension to the adjacent mid-ventricular segments and relative basal hypercontractility appears more consistent with a predominantly systemic catecholamine-mediated mechanism, likely related to circulating epinephrine excess. This interpretation is supported by current pathophysiological models suggesting that high epinephrine concentrations may induce a beta-2 adrenoceptor signaling switch from Gs to Gi coupling, thereby contributing to severe but reversible myocardial stunning [2,3]. Local cardiac sympathetic nerve terminal discharge may nevertheless have contributed more to myocardial vulnerability than to the regional heterogeneity of myocardial dysfunction, rather than constituting the dominant mechanism underlying the wall-motion abnormality pattern itself. Previous reports of postictal TTS support the reproducibility of this clinical scenario [6,10]. Recent data further suggest that TTS associated with neurological triggers may have distinctive clinical profiles, particularly with regard to arrhythmic complications [4]. In our patient, the only documented complication was non-sustained ventricular tachycardia, without other major rhythm disturbances or hemodynamic instability.

The biomarker profile was also consistent with TTS, as the peak high-sensitivity troponin T level remained relatively modest (140 pg/mL) despite profound but reversible left ventricular systolic dysfunction (LVEF 20%-25%), consistent with the well-recognized disproportion between biomarker release and the extent of ventricular dysfunction in TTS [1-3]. Moreover, the subsequent rapid decline in troponin levels was compatible with a washout phase after acute but limited myocardial injury and paralleled the early functional recovery, further supporting transient myocardial stunning rather than extensive ischemic necrosis [1-3].

The main feature of this case is recurrence after 11 months with documented phenotypic switching. The first episode, in November 2024, corresponded to a mid-ventricular variant, whereas the recurrent episode showed an apical phenotype extending to the mid-ventricular segments. Studies on TTS recurrence have shown that successive episodes may adopt different morphologies, even in the same patient [7]. The case reported by Saito et al. is particularly relevant, as it illustrates short-term recurrence with phenotypic variation [8]. Other reports have also emphasized the morphological diversity of recurrent TTS and the possible influence of neuropsychiatric comorbidities [11-13]. Our case, therefore, reinforces the concept that susceptibility to TTS may persist over time while manifesting differently at the segmental level.

Consensus and expert documents emphasize the importance of a multimodality approach to TTS [1,2]. Coronary angiography remains essential to exclude an acute culprit lesion, even when pre-existing non-obstructive coronary artery disease coexists [2]. In our patient, the coronary angiogram performed during the second episode was unchanged compared with the previous examination, supporting the absence of a new acute coronary mechanism.

CMR imaging also contributed substantially to diagnostic confirmation. Performed on day 3, it showed normalized wall motion with persistent mild residual myocardial edema. This pattern supported the diagnosis of TTS in the subacute recovery phase and argued against active myocarditis, without claiming to exclude it absolutely. The dissociation between rapid mechanical recovery and persistence of a subtle tissue abnormality represents an important teaching point of this case.

The functional recovery observed as early as day 3, with improvement in LVEF from 20%-25% to 52%, was particularly striking. This finding highlights the marked reversibility of TTS, including in severe forms [1,3]. However, rapid recovery should not obscure the potential severity of the syndrome. TTS may be associated with arrhythmic complications, particularly in forms related to neurological disorders [4]. More severe presentations, including cardiogenic shock triggered by central neurological lesions, have also been described [14]. In our case, the introduction of a beta-blocker was prompted by the documented ventricular arrhythmia and was intended to reduce arrhythmic risk.

## Conclusions

This case illustrates recurrent seizure-triggered TTS with documented phenotypic switching from a first mid-ventricular episode to a second predominantly apical episode with mid-ventricular extension. The close temporal relationship between the generalized tonic-clonic seizure and symptom onset, the profound but rapidly reversible left ventricular dysfunction, the relatively modest troponin peak, and the CMR findings strongly supported postictal TTS rather than acute myocardial infarction or active myocarditis.

This observation also emphasizes that seizure-triggered TTS should not be approached as an isolated cardiac event, but rather within a multidisciplinary neuro-cardiac framework. In our case, the marked cardiac consequences of the seizure led to reassessment of the antiepileptic regimen, supporting the concept that improved seizure control may help reduce future autonomic surges and potential myocardial recurrence. In patients with epilepsy presenting with postictal chest pain, TTS should be considered within a coordinated diagnostic and therapeutic strategy involving both cardiology and neurology, particularly in the setting of recurrence and phenotypic variability.
